# Systemic Urate Deposition: An Unrecognized Complication of Gout?

**DOI:** 10.3390/jcm9103204

**Published:** 2020-10-03

**Authors:** Puja Khanna, Richard J. Johnson, Bradley Marder, Brian LaMoreaux, Ada Kumar

**Affiliations:** 1 Division of Rheumatology, Department of Medicine, University of Michigan, Ann Arbor, MI 48109, USA; 2 Division of Renal Diseases and Hypertension, University of Colorado, Aurora, CO 80045, USA; richard.johnson@ucdenver.edu; 3 Horizon Therapeutics plc, 150 S. Saunders Rd, Lake Forest, IL 60045, USA; bmarder@horizontherapeutics.com (B.M.); blamoreaux@horizontherapeutics.com (B.L.); akumar2@horizontherapeutics.com (A.K.)

**Keywords:** gout, uric acid, systemic deposition of urate

## Abstract

Gout, an inflammatory arthritis, affects over nine million people in the US with increasing prevalence. Some medical societies do not recommend treating gout unless it is recurrent. While soft tissue urate deposits (tophi), resultant bone erosions, and joint inflammation are frequently recognized in gout, urate crystal deposits in other sites have been thought to be rare. Recent diagnostic testing, such as dual energy computed tomography (DECT), has led to the recognition that urate deposits are not uncommon in other tissues including the vasculature. To understand the potential risks for untreated gout, we reviewed the literature on extra-articular urate deposition documented by autopsy, histopathology, surgery, and radiology, including the heart, blood vessels, kidney, spine, eye, skin, and gastrointestinal system. These studies extend the significance of gout beyond the rheumatologist and emphasize the need for physicians to follow the American College of Rheumatology guidelines to treat subjects with gout to a goal of achieving serum urate <6 mg/dl. Given the growing body of literature on extraarticular urate deposition, further studies and clinical trials are needed to determine the clinical consequences of systemic urate deposition, including if reducing cardiac and vascular urate deposits may provide a survival benefit for this at-risk population.

## 1. Introduction

Gout is the most common inflammatory arthropathy in the United States, affecting nearly nine million people, and the prevalence increased by 7.2% from 1990 to 2017 [1]. Typically, it is viewed as a painful arthritis due to urate crystal deposition which can be treated intermittently with anti-inflammatory agents, and some medical societies recommend initiating urate lowering therapies only when repeated flares occur [2]. While it is widely recognized that urate crystals can deposit in the periarticular soft tissues (tophi) and joints, the concept that monosodium urate (MSU) may deposit in other extra-articular sites has generally been considered exceptionally rare and of little clinical significance. 

Recent studies suggest that peripheral and cardiovascular deposition of uric acid crystals is much more common than previously known [3]. Systemic deposition of urate and its resultant chronic inflammation may be the potential link to the frequent comorbidities associated with gout. For example, a 2007–2008 NHANES survey demonstrated that, among gout patients, 74% had hypertension, 71% had chronic kidney disease (stage >2), 26% had diabetes, 14% had a history of a myocardial infarction, 11% had heart failure, and 10% had a history of stroke. Furthermore, the greater the severity of hyperuricemia, the greater prevalence of these co-morbidities [4]. In one analysis, gout patients had an average of four co-morbidities, with 10% having seven or more co-morbidities [5,6]. Despite known associations of gout with renal disease, cardiovascular disease and metabolic syndrome, a causal link has yet to be established [7]. There is evidence that the presence of subcutaneous tophi is an independent predictor of both cardiovascular and non-cardiovascular causes [8]. When evaluating articular tophus volumes, total tophus volumes measured on dual energy computed tomography (DECT) were significantly correlated with the Framingham risk score and the number of metabolic syndrome components in patients with gout. In addition, patients with positive DECT results demonstrated significantly higher systolic and diastolic blood pressure, fasting glucose levels, and a higher prevalence of chronic kidney disease compared to those with a negative DECT study [9]. 

It is known that many animals that develop gout, such as birds and reptiles, can develop crystalline deposits in the blood vessels, heart, and other organs (visceral gout). Humans, however, were thought not to develop this complication, at least commonly. However, the study by Klauser et al. noted that 86% of gout patients had vascular deposits on DECT of the chest, including 32% having coronary artery urate deposits [3]. 

Here, we review the literature for extra-articular deposition of urate to summarize the sites where urate deposition is occurring. Subclinical urate deposition could potentially cause chronic local and systemic inflammation which may contribute to the development and progression of commonly associated co-morbidities in patients with gout. Given that systemic extra-articular urate deposition may not be as rare as originally considered, these findings would support the American College of Rheumatology recommendations for a treat-to-target management strategy with a goal of achieving serum uric acid levels <6 mg/dL [10].

## 2. Methods

PubMed from 1920 to 2020 was searched to identify reports of systemic urate deposition outside of the extremities. The search terms included extra-articular gout, systemic deposition of urate, ocular gout, gout nephropathy, renal tophi, gouty heart, cardiac valves and urate, urate deposition in the arteries, prostate and urate, autopsy findings in gout, cutaneous urate deposits, gouty panniculitis, auricular gout, breast and urate, gastrointestinal gout, pancreas and tophus, laryngeal tophus, and spinal gout. The reference lists from these publications were used to identify additional articles. The abstracts were reviewed to determine their relation to the topic, suitability, and quality. Articles were limited to the English language and only included reports in humans. Altogether 290 articles were identified and included published abstracts, case reports, and case series. The articles were reviewed for organ system involvement, and documented based on sites of urate deposition within an organ system and the diagnostic techniques used (Table 1).

## 3. Results

### 3.1. Cardiovascular

Numerous studies have shown that gout is strongly associated with an increased risk of hypertension, cardiovascular disease and stroke [9,11,12]. The etiology of this association, however, has not been fully elucidated. There were 21 articles reporting urate deposition in the cardiovascular system (Table 1). 

In the myocardium and endocardium, MSU deposits have been found on autopsy and histopathology. One autopsy reported extensive tophaceous material in the myocardial interstitium extending into the epicardial fat [13]. Another autopsy reported tophi within the cardiac conduction pathway in a patient with a history of complete heart block [14]. A case reported a gout patient presenting with acute myocarditis whose endomyocardial biopsy revealed MSU deposits with adjacent inflammatory cells. 

**Figure 1:**
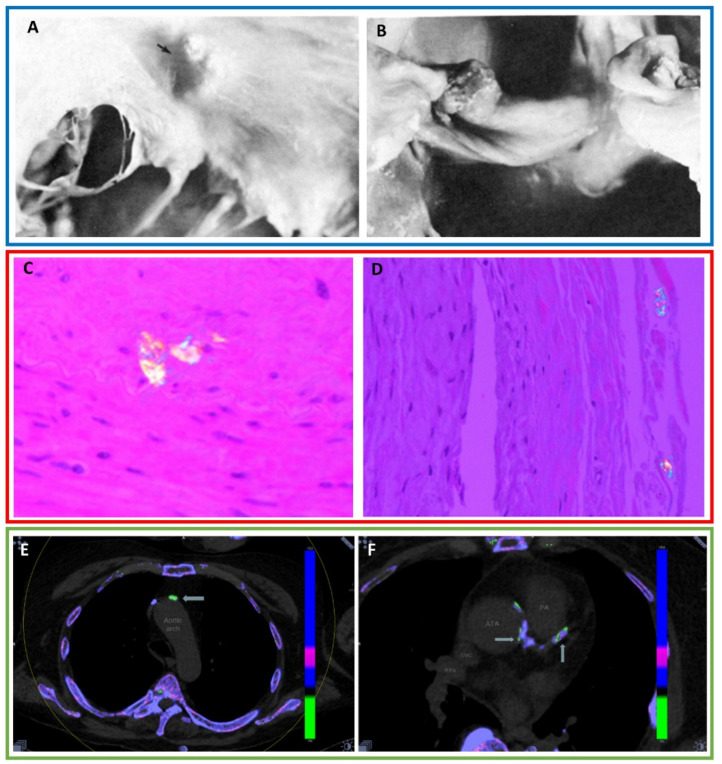
(**A**) Photograph from necropsy of a mitral valve showing a tophaceous nodule (black arrow). (**B**) Photograph from necropsy of a pulmonic valve demonstrating a tophaceous nodule. With permission from Curtiss EI, et al., Pulmonic regurgitation due to valvular tophi. *Circulation* 1983;67(3):699–701, https://www.ahajournals.org/doi/10.1161/01.CIR.67.3.699, Copyright ©1983 by American Heart Association [18]. The Creative Commons license does not apply to this content. Use of the material in any format is prohibited without written permission from the publisher, Wolters Kluwer Health, Inc. Please contact permissions@lww.com for information. (**C**) Histopathology of the left anterior descending artery reveals a microtophus. (**D**) Polarizing light microscopy image of negatively birefringent crystals in the adventitia of the right coronary artery. With permission from Park, J.J., et al., Prevalence of birefringent crystals in cardiac and prostatic tissues, an observational study. *BMJ Open* 2014, 4:e005308, distributed in accordance with CC BY-NC 4.0, https://creativecommons.org/licenses/by-nc/4.0/ [19]. (**E**) DECT of the chest reveals 20 mm^2^ of uric acid in the aortic arch (arrow). (**F**) DECT of the chest reveals several areas of urate deposition within the ascending thoracic aorta (ATA) and the coronary artery (arrows). With permission from Barazani S., et al. Detection of Uric Acid Crystals in the Vasculature of Patients with Gout Using Dual-Energy Computed Tomography (abstract). *Arthritis Rheum.* 2018;70 (suppl. 10) https://acrabstracts.org/abstract/detection-of-uric-acid-crystals-in-the-vasculature-of-patients-with-gout-using-dual-energy-computed-tomography/. ©Copyright 2018 American College of Rheumatology [22]. Images from a single patient are outlined in a box.

Nine publications have reported urate deposition on the cardiac valves detected by autopsy, histopathology, and transthoracic echocardiograms (Figure 1A,B) [15,16]. MSU deposition has been reported on all cardiac valves: mitral (6), tricuspid (1), aortic (1), and pulmonic (1) [13,17,18]. In all cases, the patients had a history of tophaceous gout. A heart murmur was present in four cases.

Urate deposits within the arterial walls and the arterial lumen have been seen on autopsy, histopathology and imaging. The first published report was an autopsy of a patient with tophaceous gout where birefringent crystals were found within the connective tissue of a coronary artery that had marked intimal wall thickening resulting in significant luminal narrowing [14]. Microtophi have been seen in the walls of the coronary arteries including the intima of the left anterior descending artery and adventitia of the right coronary artery (Figure 1C,D) [19]. Carotid endarterectomy and aortic aneurysm specimens have demonstrated intraluminal uric acid crystals which were adjacent to cholesterol deposits in atherosclerotic plaque [20,21]. 

More recently, DECT has demonstrated the ability to visualize MSU deposition within atherosclerotic plaque [22]. A prospective DECT study reported MSU deposits in the neck and chest vasculature including the coronary arteries in 88% of non-tophaceous and tophaceous gout patients (Figure 1E,F) [22]. A second prospective study performing DECT of the chest demonstrated vascular urate deposition including within the coronary arteries in 86.4% of gout patients compared with 14.9% of non-gout controls. This study also performed cadaveric chest DECT examinations and regions positive for MSU were biopsied. Polarized light microscopy confirmed MSU deposits in 7/8 biopsy specimens yielding an 87.5% positive predictive value of DECT for detection of vascular urate deposition [3]. A retrospective DECT study assessing the coronary arteries for urate deposition found that 85% of tophaceous gout patients had intraluminal coronary artery MSU deposits compared with 2% in non-gout controls [23]. 

Atherosclerosis is a dynamic process which includes lipid deposition, endothelial damage and infiltration of leukocytes and macrophages stimulated by inflammation. We theorize that intraluminal arterial urate deposition may promote atherogenesis by a proinflammatory effect. In addition, MSU deposits could incite a local inflammatory response within adjacent calcified plaque potentially making these plaques more susceptible to rupture, and thereby increasing cardiac risk. The clinical ramifications of urate deposits admixed with calcified atherosclerotic plaque remain undetermined, and may provide valuable insight into a possible link between gout and cardiovascular diseases. Since, currently, there is a lack of data in identifying patients who may be at risk for cardiovascular urate deposition, further studies examining which patients may be susceptible for systemic urate deposition and whether lowering sUA to treat-to-target levels may modify adverse cardiovascular outcomes in gout patients are warranted.

### 3.2. Renal

Historically termed “gouty nephropathy”, urate deposition has been reported within the kidneys. There are 25 articles reporting urate deposition in the renal parenchyma (exclusive of nephrolithiasis) through autopsy, histopathology and imaging (Table 1). 

**Figure 2:**
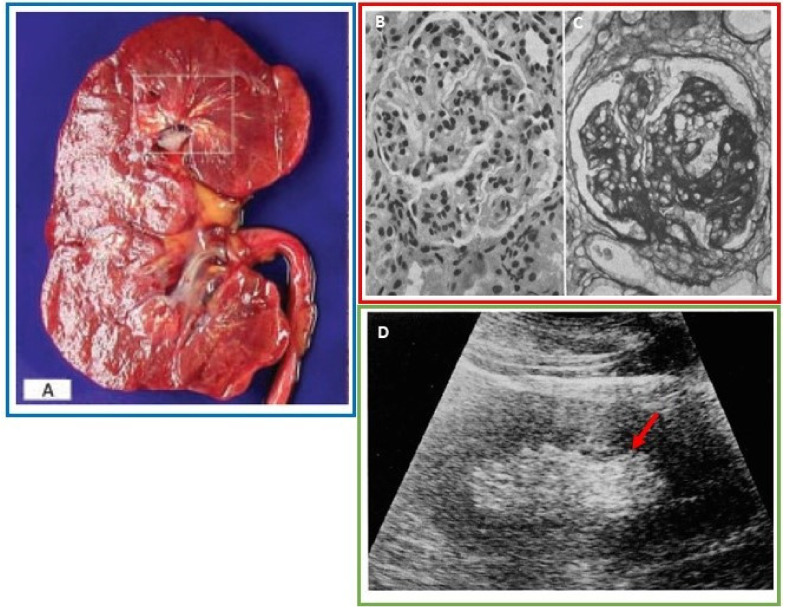
(**A**) Thin, linear, yellowish urate deposits within the renal medulla and pyramids. Reprinted from Incontinence & Pelvic Floor Dysfunction; Hsu, Y.H.; Chronic Urate Nephropathy; 2012; 6(3):89 with permission from the *Official Journal of the Taiwanese Continence Society and Taiwan Urogynecology Association* [31]. (**B**) Hematoxylin and eosin (H&E) stained section (original magnification ×575) reveal moderate glomerulosclerosis with prominent nuclei, thickened capillary walls, and prominent capillary epithelial cells [34]. (**C**) Periodic acid-Schiff (PAS) stained section (original magnification ×575) reveal advanced glomerulosclerosis with more prominent thickening of the capillary walls and axillary stroma. With permission from *Annals of Internal Medicine*, Gonick, H.C., et al. The Renal Lesion in Gout. 1965;62:667–74. Copyright©1965 American College of Physicians. All rights reserved. Reprinted with the permission of American College of Physicians, Inc. [34]. (**D**) Renal ultrasound demonstrates increased echogenicity within the renal medulla (red arrow). Adapted with permission from Kim, M.Y. *J. of Korean Radiol. Soc.* 1994 Sep;31(3):523–527. Copyright ©The Korean Radiological Society [37]. Images from a single patient are outlined in a box.

In almost all reports, urate deposition was seen in the renal medulla both within the collecting ducts and medullary interstitium [24,25,26,27,28,29,30,31] (Figure 2A). It is postulated that uric acid initially precipitates in the collecting tubules secondary to their low pH environment. As the collecting tubular walls incur damage and degrade from uric acid induced inflammation, the crystals erode through the tubular basement membrane and into the medullary interstitium. The alkaline pH and high sodium concentration milieu of the medulla transforms uric acid to MSU with formation of microtophi [32]. On gross pathology, renal urate deposits are described as thin yellowish chalk-like streaks extending through the renal medulla to the tips of the papilla [29]. On histopathology, the collecting tubules showed dilatation, hyaline degeneration and distortion. The tubules were also reported to be filled with crystals and inflammatory cells. On autopsy, the kidney sizes varied from normal to atrophic. On post-mortem examination, loss of the normal corticomedullary differentiation has been reported [27,30,33]. The renal capsular surfaces have been described as rough and granular with areas of cortical scarring [30]. Cortical thinning/scarring may be secondary to glomerular atrophy and necrosis from prolonged tubular obstruction and fibrosis secondary to chronic inflammation. 

In 89% of cases, urate deposits were surrounded by numerous inflammatory cells including lymphocytes, mononuclear inflammatory cells and giant cells. Adjacent to, and intermingled with peri-tubular inflammation were regions of tubulointerstitial fibrosis presumably secondary to chronic inflammation [25,26,29,34,35]. 

Renal vasculature involvement including nephrosclerosis, glomerulosclerosis and arteriosclerosis were reported in 74% of cases by histopathology and autopsy (Figure 2B,C). Hyaline degeneration and intimal thickening of the small and medium sized arteries, as well as the arterioles, with occlusions/near occlusions of the arterioles, were commonly found [27,28,29,30,34,35]. Medial thickening and prominent subintimal collagen have also been frequently reported in the interlobar arteries [36]. 

Four publications reported abnormal hyperechoic renal medullas on ultrasound in gout patients [37,38,39] (Figure 2D). In one retrospective review, the severity of the abnormal ultrasound findings including renal medullary echogenicity as well as renal cortical deformity correlated with increasing sUA levels [37]. In a prospective study, 36% of gout patients had diffuse hyperechoic renal medullas, and patients with abnormal renal ultrasounds had a longer duration of gout with 93% reporting articular tophi [38]. 

Urate nephrolithiasis is seen in approximately 10–20% of gout patients [40]. DECT can evaluate the composition of renal calculi including urate nephrolithiasis [41]. Distinguishing urate calculi from calcium, struvite and cystine stones has therapeutic implications since urate calculi are often treated by alkalinizing the urine.

Gout patients often have concomitant renal disease [42] and elevated sUA levels are more common in patients with chronic kidney disease (CKD). While one explanation may be that renal dysfunction impairs renal excretion of uric acid, experimental evidence in rat studies suggests that elevated sUA induces oxidative stress and endothelial dysfunction resulting in glomerular hypertension with increased vascular resistance and reduced renal flow [43,44]. Hyperuricemia induced in these rats demonstrated cellular changes associated with renal fibrosis notable in CKD [45]. Furthermore, studies indicate that lowering uric acid levels in diabetic mice ameliorates tubulointerstitial injury [46]. Based on this evidence, we hypothesize direct urate deposition and subsequent local inflammation in the renal parenchyma coupled with vascular pathology may be a source of ongoing subclinical renal damage contributing to the development and progression of CKD.

### 3.3. Spine

**Figure 3:**
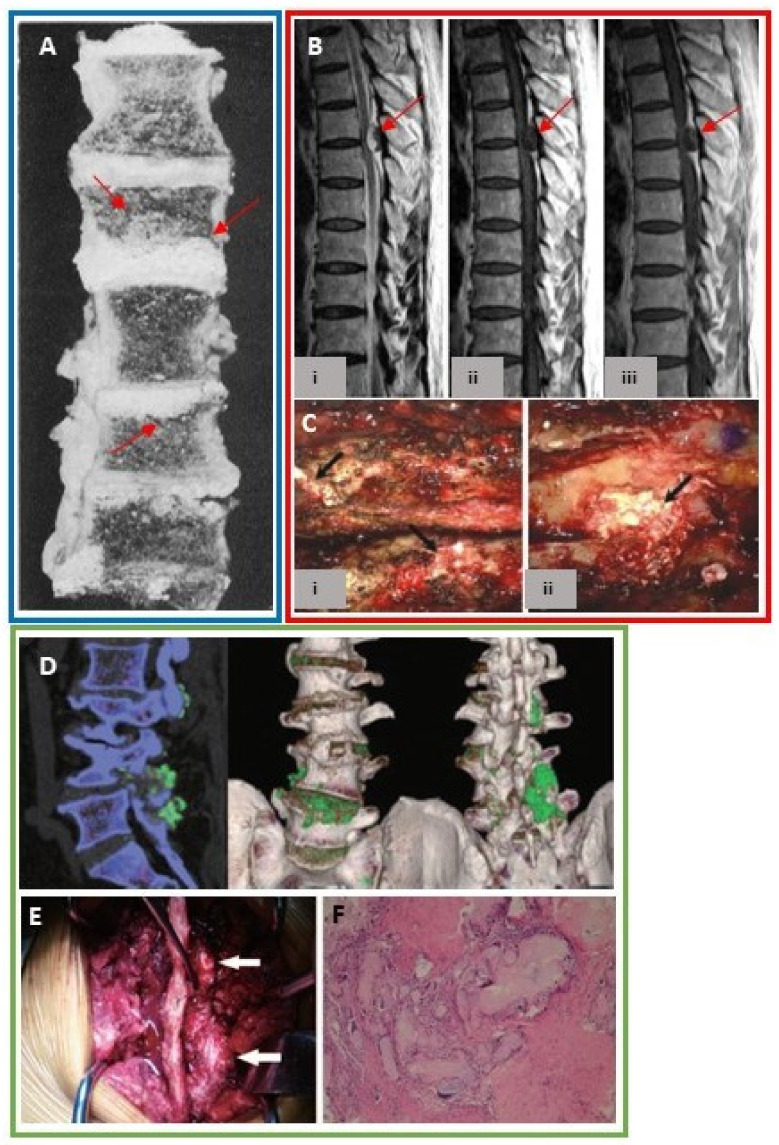
(**A**) Cross section of the spine from necropsy demonstrates urate deposits within the intervertebral discs with extension into the adjacent vertebral bodies (red arrows). With permission from an article published in *Am. J. Pathol.* 1956;32(5):871–895, Lichtenstein L., et al., Pathologic changes in gout; survey of eleven necropsied cases, copyright Elsevier 1956 [27]. (**B**) Sagittal (i) T2-weighted (ii) T1-weighted (iii) T1-weighted post gadolinium MRI of the thoracic spine shows an epidural mass (red arrows) compressing the spinal cord [48]. (**C**) Intra-operative findings on the same patient reveal a chalky whitish mass within the (i) epidural space under the ligamentum flavum extending into the T5-T6 neural foramina and (ii) T5-T6 and T6-T7 interlaminar space. Histopathology reveal urate crystals. With permission from Yoon J-W, et al, Tophaceous Gout of the Spine Causing Neural Compression. *Korean J. Spine* 2013;10(3):185–188 distributed in accordance with CC BY-NC 3.0, http://creativecommons.org/licenses/by-nc/3.0/ [48]. (**D**) Sagittal and 3-dimensional reconstruction DECT images of the lumbar spine display urate deposits (green) within L1-L5 intervertebral discs. Tophi are also seen within the L2-L3 and L4-L5 facet joints [51]. (**E**) Intra-operative findings in the same patient demonstrate chalky white material at L2-L3 and L4-L5 facet joints corresponding to the DECT images. (**F**) Histopathology confirms urate crystals. With permission from Lu H, et al. Tophaceous gout causing lumbar stenosis: A case report. *Medicine* (Baltimore) 2017;96(32):e7670. https://journals.lww.com/md-journal/Fulltext/2017/08110/Tophaceous_gout_causing_lumbar_stenosis__A_case.22.aspx**,** [51]. The Creative Commons license does not apply to this content. Use of the material in any format is prohibited without written permission from the publisher, Wolters Kluwer Health, Inc. Please contact permissions@lww.com for information. Images from a single patient are outlined in a box.

Over one hundred cases in the literature report urate crystal deposition in the spine, with the first case detected in 1950 [47]. Reports include autopsy, histopathology, surgical and imaging findings (Table 1). MSU deposition has been found within the cervical, thoracic, and most frequently lumbar spine [48,49]. Anatomically, facet joints are synovial joints, therefore, MSU deposition in the posterior elements of the spine represents articular and periarticular involvement. However, MSU deposition is not confined to the facet joints, but is present within the intervertebral discs (Figure 3A), interspinous ligaments, ligamentum flavum, epidural space, pedicles, lamina, and paraspinal soft tissues [48]. It is hypothesized that lower pH and temperature in the presence of underlying degenerative changes may predispose patients to spinal urate deposition [50]. 

Spinal gout may mimic other clinical conditions including degenerative disc disease and infection. The most common reported symptom was back pain which often correlated to the location of urate deposition [49]. Spinal tophi may compress the nerve root or spinal cord [48] causing neurologic impairment including symptoms of radiculopathy, myelopathy, and bowel/ bladder dysfunction [50] (Figure 3B,C). Misdiagnosis can result in unnecessary surgery and hospitalization [49,50]. Though typically considered rare, some reports estimate spinal symptoms maybe the initial manifestations of gout in 25% of patients [50]. The prevalence of spinal gout may be underestimated due to the nonspecific clinical symptoms and imaging findings. Previously, diagnosis has required spinal intervention. However, newer imaging techniques permit a non-invasive diagnosis.

Detecting spinal urate deposition by imaging is limited, since urate crystals are not radiodense, and therefore cannot be visualized on radiographs. Magnetic resonance imaging (MRI) and computed tomography (CT) findings are often nonspecific [50] though may have a similar appearance as in the extremities, with soft tissue tophi and adjacent osseous erosions especially in the posterior elements [51]. DECT has recently been used to accurately detect MSU crystals throughout the spine [52] (Figure 3D–F). A retrospective analysis of DECT examinations in gout patients found spinal urate deposition in 60% of scans (in 83% of symptomatic, and 25% of asymptomatic cases) [53]. Another prospective study of the lumbosacral spine showed gout patients had significantly higher volumes of spinal MSU deposits compared to non-gout controls and the volume of spinal MSU deposits was proportional to sUA levels [54].

Unfortunately, due to the nonspecific clinical and imaging findings, the majority of cases were misdiagnosed and therefore underwent surgery and/or biopsy. Laminectomies were the most commonly reported surgical intervention [49,50]. Studies reporting resolution of spinal symptoms after urate lowering therapy (ULT) suggest that medical management especially in gout patients should be considered in order to optimize non-invasive treatment options and potentially improve patient outcomes [50,52].

### 3.4. Ocular

Thirty-six articles were found in the literature documenting ocular urate deposition as seen by histopathology and clinical exam (Table 1). Urate deposits have been reported in nearly all ocular and adnexal structures including the eyelid [55], medial and lateral canthus [56,57], conjunctiva [58], sclera [59], cornea [60], lens, iris [61], orbital fossa [62], and retina [63]. The eyes may be predisposed to tophi secondary to lower body temperatures and a low pH environment resulting in poor solvent capabilities [61,64].

**Figure 4:**
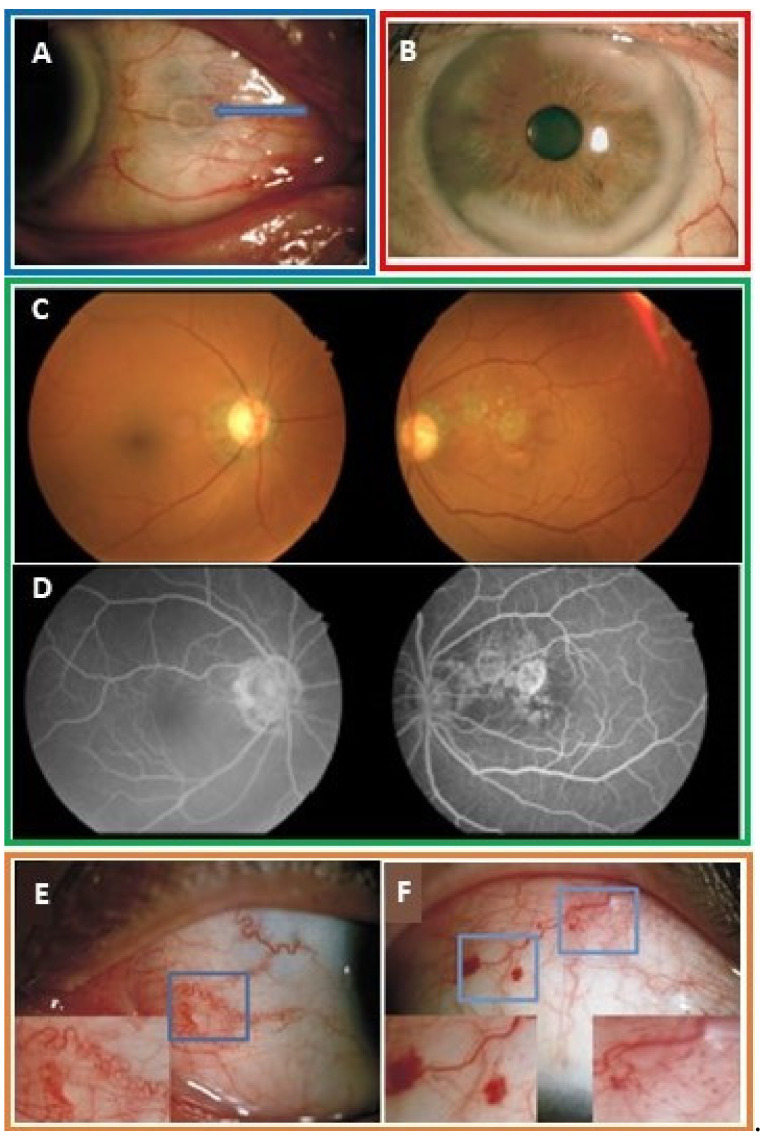
(**A**) Photograph displaying ring-like chalky white deposits along the superficial sclera. With permission from Lin, J., et al. Characteristics of ocular abnormalities in gout patients. *Int. J. Ophthalmol.* 2013;6(3):307–311 [59]. (**B**) Photograph demonstrating chalky white deposits on the corneal stroma. Corneal scraping revealed MSU crystals under light microscopy. Hyperemic episcleral and conjunctival vessels were also visible. With permission from Bernad, B. et al., Clinical image: corneal tophus deposition in gout. *Arthritis Rheum.* 2006;54(3):1025, Copyright ©2006 by the American College of Rheumatology [60]. (**C**) Fundoscopic exam images reveal numerous small refractile yellow lesions in the macula suggestive of crystal deposition as well as areas of geographic atrophy [63]. (**D**) Fluorescein angiogram images demonstrate window defects and regions of peripapillary atrophy. With permission from Jiang, Y., et al. Retinal complications of gout: a case report and review of the literature. *BMC Ophthalmol.* 2018;18(1):11, distributed in accordance with a Creative Commons Attribution 4.0 International License (http://creativecommons.org/licenses/by/4.0/ [63]. (**E**) Photograph showing tortuous blood vessels along the scleral and conjunctival surfaces. (**F**) Photograph shows multiple subconjunctival hemorrhages. With permission from Lin, J., et al. [59]. Images from a single patient are outlined in a box.

Urate deposition in the sclera and episclera may manifest as anterior and posterior scleritis, tenonitis or nodular and recurrent episcleritis [64]. Scleral tophi may present as chalky white lesions on the scleral surface (Figure 4A) [59]. Tophi have been seen in all layers of the cornea including the corneal epithelium, stroma and Bowman’s layer and may lead to corneal ulcers (Figure 4B) [60,64]. MSU deposits in the iris and the anterior chamber have been reported as clear, gelatinous deposits on the surface of the iris and anterior chamber angle [59,61]. There are several case reports of biopsy-proven conjunctival and subconjunctival MSU deposits [58,65,66]. A single case report documented a nonspecific orbital mass within the intra-orbital fossa which was biopsy proven to be tophus. Another case report described subretinal crystal deposits with adjacent regions of macular atrophy on fundoscopic exam and fluorescein angiogram in a patient with tophaceous gout presumed to be urate crystals [63] (Figure 4C,D). Recurrent uveitis has been seen in gout patients and may resolve with colchicine and corticosteroids [64].

Red eye, the most common ocular symptom in gout patients, may be partially attributed to hyperemic conjunctival and episcleral vessels [67]. Conjunctival vessels may become markedly tortuous, dilated, and fragile making them susceptible to subconjunctival hemorrhage [59] (Figure 4E,F). Transparent conjunctival vessels have been reported to be four times more common in gout patients compared with controls [59]. These vascular changes may be indicative of an underlying urate induced microvascular disease, though the exact pathophysiology of the vascular fragility is not known [68].

### 3.5. Gastrointestinal

Urate deposition has been demonstrated in the gastrointestinal system through imaging, autopsy, and histopathology (Table 1). 

Liver: There are two case reports of hepatic tophi. Intra-hepatic tophi mimicked malignancy on imaging warranting biopsies, which revealed MSU crystals [69]. 

Pancreas: Four case reports documented biopsy-proven tophi within the pancreas [70,71]. Intra-pancreatic tophi were indistinguishable from malignancy and/or pancreatic pseudocysts on imaging. In two cases, pancreatic tophi completely resolved with ULT. 

**Figure 5:**
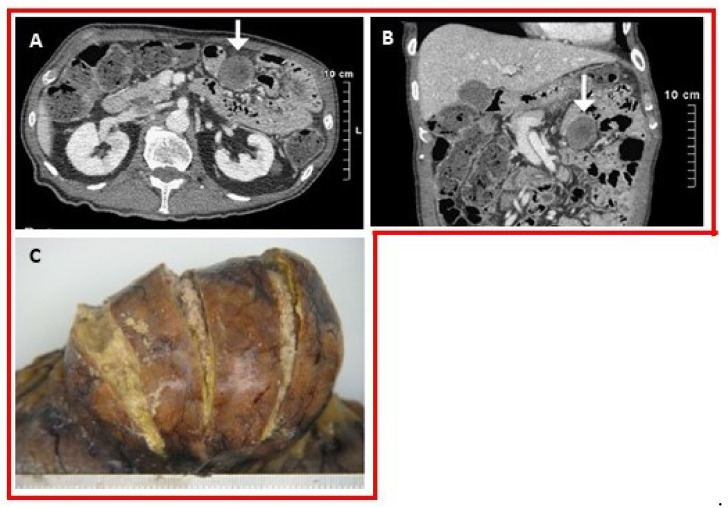
(**A**) Axial and (**B**) coronal images of a contrast-enhanced abdominal CT reveals a mass arising from the small bowel (white arrow) [76]. (**C**) Photograph of the resected specimen demonstrates a subserosal mass arising from the small bowel with yellowish material emanating from the surface. Histopathology revealed a cystic mass whose contents contained MSU crystals [76]. Images from a single patient are outlined in a box. Reprinted from *Human Pathology: Case reports* 2014;1:2–5 Katoch et al. Small intestinal tophus mimicking tumor, with permission from Elsevier. ©2014 Elsevier Inc. [76].

Bowel: Six case reports documented biopsy-proven MSU within the small intestine, large intestine and submucosal/mesenteric surfaces of the small bowel. In two autopsy reports, the patients had a long history of tophaceous gout and multiple tophi were found along the mesenteric surfaces of their small intestines [72,73]. In one patient, abnormal blunting and distorted shapes of the jejunal villa was seen on histopathology [72]. A submucosal bowel tophus was discovered in a patient who presented with a sigmoid bowel perforation (Figure 5A,B). Postoperatively, urate crystals were discovered in the surgical draining tubes [74]. There were two case reports of subserosal tophi within the jejunum and transverse colon (Figure 5C) [75,76]. 

Intra-abdominal: There is a single article reporting an intra-pelvic mass mimicking an abscess on imaging, which on aspiration yielded urate crystals [77].

### 3.6. Integumentary

**Figure 6:**
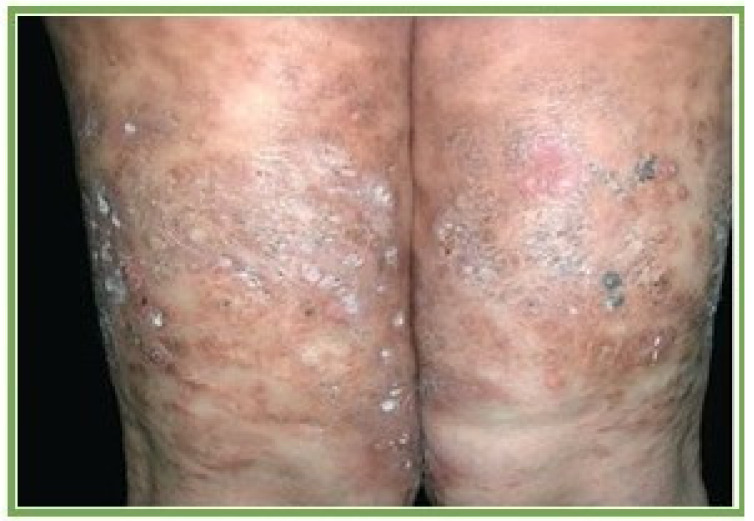
Cutaneous nodules and plaques. With permission from Pattanaprichakul, P., et al. Disseminated gouty panniculitis: an unusual presentation of extensive cutaneous tophi. *Dermatol. Pract. Concept* 2014;4(4):33–35, distributed in accordance with a Creative Commons Attribution license, http://creativecommons.org/licenses [78].

Miliarial gout, gouty panniculitis and gout nodulosis are terms used to describe the cutaneous manifestations of intradermal MSU deposits. It is hypothesized that uric acid may deposit in regions of previous tissue damage [78]. There were 48 articles documenting urate deposition in the integumentary system demonstrated by autopsy, histopathology, and clinical exam (Table 1). Dermal tophi often presented as subcutaneous nodules or indurated plaques which may ulcerate (Figure 6). In some cases, a chalky substance drained from the ulcer. Skin involvement occasionally preceded articular symptoms [79]. On histopathology, inflammatory cells were often seen adjacent to MSU deposits. Dilated blood vessels within the dermis as well as wall thickening of the small and larger dermal vessels have been reported under light microscopy [80]. Gouty panniculitis is secondary to MSU deposits in the lobular hypodermis [81], and in these cases, histopathology revealed urate crystals with adjacent fat lobular inflammation secondary to lymphocytic infiltration [78]. In many of the reported cases, ULT resolved existing skin lesions and may have prevented new lesion formation [78,82]. 

### 3.7. Head and Neck

Twenty-one publications reported MSU deposition within the larynx, middle ear [83], Eustachian tube surface [84], and nose through histopathology, clinical exam, and imaging (Table 1). There were six cases of middle ear involvement with the most common clinical presentation being conductive hearing loss and otorrhea [83,85]. In almost all cases, abnormal otoscopic findings prompted a CT scan which revealed a nonspecific soft tissue mass in the middle ear with adjacent bone erosion/destruction [83]. In all cases, surgery was performed and MSU deposits were confirmed on histopathology. Interestingly, none of these patients had a history of gout. There were eleven cases of urate deposition involving the larynx including the vocal cords, subglottis, and the cricoarytenoid joint [86,87,88,89]. These patients often presented with hoarseness, odynophagia, dysphagia or stridor [89]. Four cases of nasal MSU deposits have been reported manifesting as a nasal mass, which in some cases led to osseous destruction [90].

### 3.8. Other 

MSU crystals have been documented in the prostate gland by autopsy and histopathology as reported in two articles (Table 1) [27,91]. A proposed mechanism for prostatic urate deposition is via urinary reflux [91]. Urate crystals have been found within dilated ducts of the prostatic lumen surrounded by foreign body giant cells [27]. In one study, 48% of examined prostate glands being resected for prostate cancer in patients without gout had MSU deposits in the glandular lumen [19]. Symptoms of chronic prostatitis were also commonly reported in patients with prostatic urate deposition [91]. A study treated patients with non-bacterial prostatitis with allopurinol for three months and found a significant positive effect on patient symptoms, as well as urine urate and expressed prostatic secretion of urate [92].

**Figure 7:**
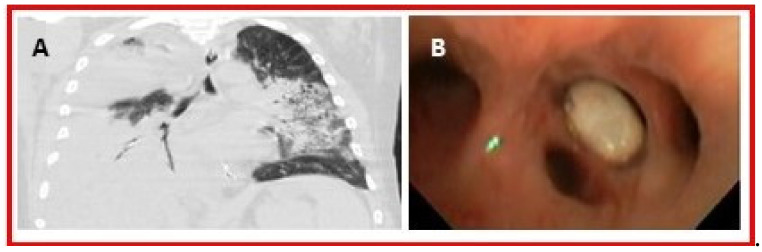
(**A**) Coronal CT of the chest reveals an endobronchial lesion within the right lower lobe bronchus. There is subsequent collapse of the right lower lobe. Other regions of multifocal consolidation are incidentally noted. (**B**) Photograph obtained during bronchoscopy reveals a mass in the right lower lobe bronchus. Histopathology revealed a tophus containing multiple MSU crystals. Adapted with permission of the American Thoracic Society. Copyright © 2020 American Thoracic Society. All rights reserved. Adamson et al. 2013. Tophus causing bronchial obstruction. *Am. J. Respir. Crit. Care Med.*;188(12):e72–e73. *The American Journal of Respiratory and Critical Care Medicine* is an official journal of the American Thoracic Society. Readers are encouraged to read the entire article for the correct context at doi: 10.1164/rccm.201301-0097IM. The authors, editors, and The American Thoracic Society are not responsible for errors or omissions in adaptations [94].

Five cases in the literature described mammary gout which presented as non-specific masses on mammography and biopsy proven to be tophi [93] (Table 1). Four articles reported urate deposition within the pulmonary system through histopathology and imaging (Table 1) including tophi presenting as lung nodules and an endobronchial mass (Figure 7A,B) [94]. In one patient, uric acid crystals were found in pleural effusions upon aspiration [95]. There was a single case report of multiple penile lesions determined to be tophi after clinical exam, surgery, and histopathology [96] (Table 1). A single case report also diagnosed a periungual tophus in the cuticle through clinical exam and histopathology [97] (Table 1).

## 4. Discussion

Gout typically has been considered a relatively benign condition associated with recurrent arthritis due to the deposition of intraarticular urate crystals. While some subjects develop destructive lesions, such as bone erosions and cartilage injury, which can permanently damage the joints, many patients are treated symptomatically with nonsteroidal agents or colchicine and do not receive urate lowering therapies. Indeed, some medical societies such as the American College of Physicians have recommended to initiate ULT and lower sUA levels only in subjects with recurrent gout with a treat-to-management strategy, while the American College of Rheumatology recommends treating subjects with gout with a treat-to-target strategy maintaining sUA levels <6 mg/dL [10].

A growing body of literature, however, suggests that chronic gout may not be as benign as originally thought, but that occult uric acid crystal deposition can occur in multiple extra-articular sites raising the important possibility that gout is really a systemic disease that can affect multiple organ systems. For example, the DECT and histopathology findings showing urate can deposit in the coronary arteries and other major arteries suggest it may have a casual role in the development and progression of cardiovascular disease. Indeed, hyperuricemia was shown to be an independent and causal risk factor for cardiovascular disease including sudden cardiac death that was confirmed by Mendelian randomization studies [98]. 

For the clinician, these studies should raise awareness that urate crystals can deposit in multiple sites, and can manifest as back pain (spinal gout), vascular or cardiac disease, or even ocular symptoms. Silent urate deposition may serve as the nidus for subclinical local inflammation in addition to chronic systemic inflammation. Treating all subjects with gout seems prudent, and we would recommend following the ACR guidelines. Most importantly, we recommend further studies using special imaging such as DECT to determine the frequency and consequences of visceral gout in humans.

Given the strong association of gout with various co-morbidities including cardiovascular disease, hypertension, CKD, diabetes, and metabolic syndrome, further investigations are warranted to determine the frequency and clinical significance of systemic urate deposition and potentially implicate urate deposition as having a causal role in these co-morbid disease processes. 

## Figures and Tables

**Table 1:** Sites of extraarticular urate deposition.

Organ System	Number of Articles	Diagnostics
Total	290	Clinical exam, surgery, autopsy, histopathology and/or imaging
Spine: Lumbar, thoracic, cervical: facet joint, lamina, pedicle, intervertebral disc, ligaments, ligamentum flavum, epidural space, paraspinal soft tissue	113	Surgery, autopsy, histopathology, imaging
Integumentary: Dermis, hypodermis	48	Clinical exam, autopsy, histopathology
Ocular: Periocular soft tissues, canthi, conjunctiva, sclera, cornea, lens, orbital fossa, retina, iris	36	Clinical exam, histopathology, imaging
Renal: Renal parenchyma (Cases of nephrolithiasis were excluded)	25	Autopsy, histopathology, imaging
Cardiovascular: Valves (mitral, pulmonic, aortic, tricuspid), myocardium, conduction pathway, coronary artery wall, arterial atherosclerotic plaque	21	Surgery, autopsy, histopathology, imaging
Larynx: Vocal cords, subglottis, cricoarytenoid joint	11	Clinical exam, histopathology
Bowel: Small intestine, colon, mesentery	6	Autopsy, histopathology, imaging
Middle Ear/ Eustachian tube	6	Clinical exam, histopathology, imaging
Breast	5	Histopathology, imaging
Pancreas	4	Histopathology, imaging
Nasal	4	Clinical exam, histopathology, imaging
Pulmonary: Bronchus, pleural effusion	4	Bronchoscopy, histopathology, imaging
Prostate Gland	2	Histopathology
Liver	2	Histopathology, imaging
Penis	1	Clinical exam, surgery, histopathology
Nailbed	1	Clinical exam, histopathology
Pelvis	1	Histopathology, imaging
